# Aqueous thermogalvanic cells with a high Seebeck coefficient for low-grade heat harvest

**DOI:** 10.1038/s41467-018-07625-9

**Published:** 2018-12-04

**Authors:** Jiangjiang Duan, Guang Feng, Boyang Yu, Jia Li, Ming Chen, Peihua Yang, Jiamao Feng, Kang Liu, Jun Zhou

**Affiliations:** 1 Wuhan National Laboratory for Optoelectronics, Huazhong University of Science and Technology, 430074 Wuhan, China; 2 State Key Laboratory of Coal Combustion, School of Energy and Power Engineering, Huazhong University of Science and Technology, 430074 Wuhan, China

## Abstract

Thermogalvanic cells offer a cheap, flexible and scalable route for directly converting heat into electricity. However, achieving a high output voltage and power performance simultaneously from low-grade thermal energy remains challenging. Here, we introduce strong chaotropic cations (guanidinium) and highly soluble amide derivatives (urea) into aqueous ferri/ferrocyanide ([Fe(CN)_6_]^4−^/[Fe(CN)_6_]^3−^) electrolytes to significantly boost their thermopowers. The corresponding Seebeck coefficient and temperature-insensitive power density simultaneously increase from 1.4 to 4.2 mV K^−1^ and from 0.4 to 1.1 mW K^−2^ m^−2^, respectively. The results reveal that guanidinium and urea synergistically enlarge the entropy difference of the redox couple and significantly increase the Seebeck effect. As a demonstration, we design a prototype module that generates a high open-circuit voltage of 3.4 V at a small temperature difference of 18 K. This thermogalvanic cell system, which features high Seebeck coefficient and low cost, holds promise for the efficient harvest of low-grade thermal energy.

## Abstract (web summary)

Achieving high thermopower in liquid-state thermogalvanic cells is vital to realize a low-cost technology solution for thermal-to-electrical energy conversion. Here, the authors present aqueous thermogalvanic cells based on modified electrolyte with enhanced Seebeck coefficient and thermopower.

## Introduction

Low-grade thermal energy (<100 °C) is ubiquitous in industrial processes, the environment, and the human body, but is mostly discarded without any effort at recovery^1–3^. Converting low-grade thermal energy into electricity is an ideal strategy for addressing global energy and environmental issues^4^. Despite the abundance of low-grade heat, harvesting energy from potential sources has proven difficult due to the irregular distribution of heat sources and very low efficiency^5^. Among thermoelectric conversion strategies, solid-state thermoelectric cells have been studied extensively in recent decades and exhibit high efficiency at high temperatures. However, harvesting low-temperature heat by using solid-state thermoelectric devices has been hindered by their high cost and the limitations of materials with toxic or rare elements^6–9^.

Liquid-state thermogalvanic cells (TGCs) offer an alternative, inexpensive, flexible, and scalable route for direct thermal-to-electric energy conversion^3^. The principal advantage of TGCs is the high Seebeck coefficient (*S*_e_) of approximately 1 mV K^−1^, which is one order of magnitude higher than that of conventional thermoelectric cells^10^. For TGC systems, the Seebeck coefficient is defined as^3^

1 $$S_{\mathrm{e}} = \Delta E/\Delta T = \Delta S/nF,$$

where Δ*E* is the open-circuit voltage, Δ*T* is the temperature difference, *n* is the number of electrons transferred in the redox reaction, *F* is Faraday’s constant, and Δ*S* is the partial molar entropy difference of the redox couple. Due to the limited temperature difference between heat sources and the surrounding ambient environment, the development of a TGC system with high *S*_e_ that can generate a high voltage even from a small temperature difference is of great importance.

Recently, increasing Δ*S* by regulating the interactions between the redox ions and solvents has enhanced Seebeck coefficients in organic electrolytes^10–16^. However, these electrolyte systems mostly suffer from low ionic conductivity and poor mass transport. Thus, their efficiencies are unsatisfactory, and the corresponding temperature-insensitive power densities (defined as the maximum power density obtained normalized to the square of the inter-electrode temperature difference, *P*_max_/(Δ*T*)^2^) are inferior to those of TGCs in aqueous electrolytes^17–19^. The simultaneous achievement of high *S*_e_ and power density in a TGC remains elusive.

Here, we introduce strong chaotropic cations (guanidinium) and highly soluble amide derivatives (urea) into the 0.4 M [Fe(CN)_6_]^3−^/[Fe(CN)_6_]^4−^ aqueous electrolyte to yield a very high *S*_e_ (4.2 mV K^−1^) and an impressive *P*_max_/(Δ*T*)^2^ (1.1 mW K^−2^ m^−2^), both of which are approximately threefold higher than the corresponding values for the pristine TGC. The results prove that guanidine chloride (GdmCl) and urea synergistically enlarge the entropy difference of [Fe(CN)_6_]^3−^/[Fe(CN)_6_]^4−^ and significantly enhance the Seebeck effect. Furthermore, we designed a module with 50 cells in series that generates an open-circuit voltage (*V*_oc_) of 3.4 V and a short-circuit current (*I*_sc_) of 1.2 mA under a small temperature difference of 18 K and could directly light a red light-emitting diode (LED) array.

## Results

### The enhancement effect of guanidinium on Seebeck coefficient

**Fig. 1:**
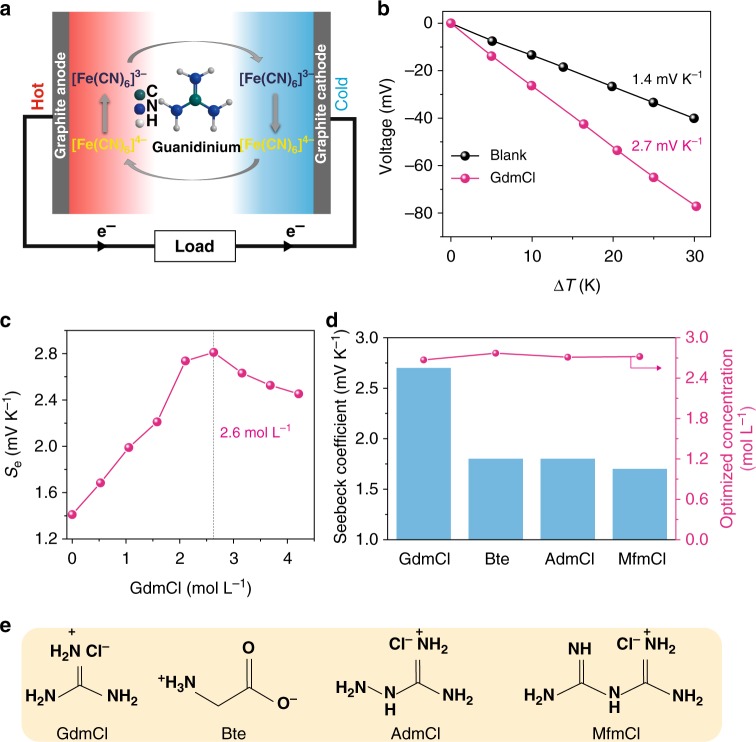
TGC containing guanidinium in 0.4 M [Fe(CN)_6_]^3−^/[Fe(CN)_6_]^4−^. **a** The schematic operation mechanism for the TGC. **b** Dependence of the open-circuit voltage for the TGC systems on the temperature difference (Δ*T*) from 0 to 30 K; blank refers to the pristine [Fe(CN)_6_]^3−^/[Fe(CN)_6_]^4−^ electrolytes without additives; GdmCl indicates the addition of guanidine chloride at the optimized concentration. **c** Dependence of the Seebeck coefficient on the concentration of GdmCl in the [Fe(CN)_6_]^3−^/[Fe(CN)_6_]^4−^ electrolyte. **d** The Seebeck coefficients for TGCs containing different chaotropic species, including GdmCl, betaine (Bet), aminoguanidine chloride (AdmCl), and metformin chloride (MfmCl), and their corresponding optimized concentrations. **e** The chemical structure of four chaotropic species

The Seebeck effect for TGCs fundamentally originates from the entropy difference of redox couples. Continuous operation of a TGC is adopted for the typical planar TGC device shown in Fig. 1a, which consists of two graphite electrodes in the K_3_[Fe(CN)_6_]/K_4_[Fe(CN)_6_] electrolyte (Supplementary Figure 1a). When a temperature gradient is built across the whole cell, the reversible reaction between the redox couple will incline to the opposite direction, thus causing a potential difference in the electrolyte at the electrodes^20^. That is, the reaction tends to the direction of the entropy increase at the hot side, and vice versa. The entropy difference of a redox couple is related to its absolute charge and reflects the strength of interactions between redox species and solvents^3,21,22^. In the ferri/ferrocyanide couple, [Fe(CN)_6_]^4−^ has a higher charge density and thus can form a more compact hydration shell (Supplementary Figures 2–4 and Supplementary Note 1); consequently, its thermodynamic entropy is lower than that of [Fe(CN)_6_]^3−^. Therefore, the oxidation of [Fe(CN)_6_]^4−^ to [Fe(CN)_6_]^3−^ occurs through the release of an electron to the electrode at the hot side, and the electron is consumed at the cold cathode through an external circuit by the reduction of [Fe(CN)_6_]^3−^ to [Fe(CN)_6_]^4−^. Based on Eq. 1, we propose that enlarging the entropy difference by regulating solvation shells of redox couples can enhance their Seebeck coefficient.

[Fe(CN)_6_]^3−^ and [Fe(CN)_6_]^4−^ are categorized as chaotropic anions in the Hofmeister series and are able to bond with chaotropic cations based on chaotrope–chaotrope ion specificity^23,24^. Therefore, strong chaotropic cations could be expected to rearrange the solvation shells of the redox couple. GdmCl is the most typical chaotrope, and its effects on water structures and protein stability have been studied extensively^25,26^. Here, we introduce GdmCl into the [Fe(CN)_6_]^3−^/[Fe(CN)_6_]^4−^ aqueous electrolyte. Figure 1b shows the temperature dependence of the cell potential over a range of Δ*T* from 0 to 30 K, and the corresponding instantaneous cell potential curves on Δ*T* are shown in Supplementary Figure 1b. The open-circuit voltages show a linear relationship with the applied temperature difference, and the corresponding *S*_e_ values are obtained from the slopes of these lines. The *S*_e_ value for the pristine electrolyte (blank in Fig. 1b) is 1.4 mV K^−1^, consistent with the reported value in the literature^27^. Surprisingly, the *S*_e_ value increases significantly to 2.7 mV K^−1^ when GdmCl is added to the pristine electrolyte. The dependence of the *S*_e_ value on the GdmCl concentration is shown in Fig. 1c. The *S*_e_ value increases with increasing GdmCl concentration and achieves a maximum of ~2.7 mV K^−1^ at a concentration of ~2.6 mol L^−1^. The enhancement effects of other chaotropic species are also assessed (Fig. 1d) and their corresponding chemical structures are shown in Fig. 1e. These additives, including betaine (Bet), aminoguanidine chloride (AdmCl), and metformin chloride (MfmCl), all increase the *S*_e_ value to ~1.8, 1.8, and 1.7 mV K^−1^, respectively. By contrast, typical kosmotropic species, including NaCl, LiCl, CaCl_2_, and MgCl_2_, barely enhance the *S*_e_ value of [Fe(CN)_6_]^3−^/[Fe(CN)_6_]^4−^ electrolytes (Supplementary Figure 5 and Supplementary Note 2). Clearly, the chaotrope–chaotrope ion specificity plays a critical role in the enhancement of the Seebeck effect of [Fe(CN)_6_]^3−^/[Fe(CN)_6_]^4−^ electrolytes. We propose that the impacts of different chaotropes on the Seebeck effect of [Fe(CN)_6_]^3−^/[Fe(CN)_6_]^4−^ electrolytes are related to their molecular size. Compared with the other chaotropes, GdmCl has the smallest size and can interact with [Fe(CN)_6_]^3−^/[Fe(CN)_6_]^4−^ most easily. Note that the optimal concentrations of the four chaotropes are all approximately 2.6–2.7 M (Fig. 1d), which is approximately equal to the total negative charge concentration (2.8 M) in the 0.4 M [Fe(CN)_6_]^3−^/[Fe(CN)_6_]^4−^ electrolytes. This result also indicates that chaotrope–chaotrope ion specificity is closely correlated with the high *S*_e_ value.

### The enhancement mechanism

**Fig. 2:**
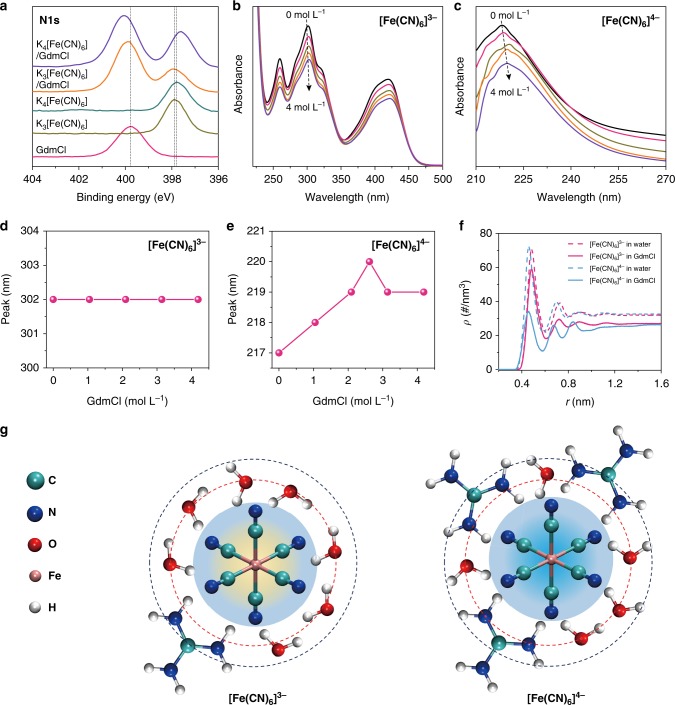
The mechanism of enhancement of the Seebeck effect by GdmCl. **a** XPS spectra shift for K_3_[Fe(CN)_6_], K_4_[Fe(CN)_6_], GdmCl, and their composites. For the composites, GdmCl at an optimized concentration of 2.6 M was added to 0.4 M K_3_[Fe(CN)_6_] or K_4_[Fe(CN)_6_]. The solid samples used for XPS measurement were prepared by drying the composite solutions in a vacuum oven at 333 K for 48 h. UV–Vis spectral shifts and the corresponding absorption peaks of [Fe(CN)_6_]^3−^ (**b**, **d**) and [Fe(CN)_6_]^4−^ (**c**, **e**) with increasing concentrations of GdmCl. **f** Radial density profiles of the hydrated [Fe(CN)_6_]^3−^ and [Fe(CN)_6_]^4−^ anions in pure water and GdmCl aqueous solution obtained from the results of MD simulation (Supplementary Note 1). **g** The schematic solvation formations of [Fe(CN)_6_]^3−^ and [Fe(CN)_6_]^4−^ in GdmCl solution

To clarify the underlying mechanism, we investigate the interaction between GdmCl and [Fe(CN)_6_]^3−^/[Fe(CN)_6_]^4−^ by X-ray photoelectron spectroscopy (XPS) and ultraviolet–visible absorption spectroscopy (UV–Vis). Figure 2a shows the N1s spectra of K_3_[Fe(CN)_6_], K_4_[Fe(CN)_6_], GdmCl, and their composites. For the mixture of K_4_[Fe(CN)_6_]/GdmCl, the N1s binding energy spectra for [Fe(CN)_6_]^4−^ and GdmCl both obviously shift compared with those of their pure species. By contrast, the N1s binding energy spectrum for K_3_[Fe(CN)_6_]/GdmCl shows little shift. Similar results are observed for the UV–Vis spectra. The absorption peak for [Fe(CN)_6_]^4−^ shifts significantly after the addition of GdmCl, and the maximum shift occurs at a GdmCl concentration of ~2.6 M (Fig. 2c, e), consistent with the optimal concentration of GdmCl for enhancing the Seebeck effect. By contrast, the absorption peak for [Fe(CN)_6_]^3−^ remains unchanged with varying concentrations of GdmCl (Fig. 2b, d). These results reveal that the guanidinium cation (Gdm^+^) has a stronger chaotrope–chaotrope interaction with [Fe(CN)_6_]^4−^ than with [Fe(CN)_6_]^3−^. This conclusion is also supported by our molecular dynamic (MD) simulations (Supplementary Note 1). The radial density profiles between the mass center of [Fe(CN)_6_]^4−^/[Fe(CN)_6_]^3−^ and a water molecule in the pristine and GdmCl systems are shown in Fig. 2f and Supplementary Figure 2. Before adding GdmCl, water molecules in the 0.4 M K_3_[Fe(CN)_6_] solution are farther away from [Fe(CN)_6_]^3−^, with a peak position at approximately 4.8 Å, whereas the peak of the radial density profile for [Fe(CN)_6_]^4−^ is approximately 4.6 Å in 0.4 M K_4_[Fe(CN)_6_] solution. The higher charge of [Fe(CN)_6_]^4−^ results in a more closely “packed” solvation shell. When GdmCl is added, due to its higher charge, Gdm^+^ has a stronger interaction with the [Fe(CN)_6_]^4−^ complex (−9161.82 kJ mol^−1^) than [Fe(CN)_6_]^3−^ (−2344.95 kJ mol^−1^). Thus, more Gdm^+^ cations are prone to bond with [Fe(CN)_6_]^4−^, resulting in greater destruction of the hydration shell. The water number density at the first peak of [Fe(CN)_6_]^3−^ decreases from 70.7 # nm^−3^ to 59.1 # nm^−3^ but from 72.7 # nm^−3^ to 33.9 # nm^−3^ for [Fe(CN)_6_]^4−^ (Fig. 2f). The schematic solvation structures of [Fe(CN)_6_]^3−^/[Fe(CN)_6_]^4−^ in GdmCl solution are illustrated in Fig. 2g. Apparently, a significant difference in solvation shells between [Fe(CN)_6_]^3−^ and [Fe(CN)_6_]^4−^ is achieved by adding GdmCl. More Gdm^+^ cations compactly surround [Fe(CN)_6_]^4−^, resulting in the rearrangement of its solvation shell. By contrast, the solvation shell of [Fe(CN)_6_]^3−^ is only slightly affected by Gdm^+^ cations. Therefore, the entropy difference of [Fe(CN)_6_]^3−^/[Fe(CN)_6_]^4−^ dramatically increases, resulting in a high *S*_e_ value.

### The synergistic effect of guanidinium and urea

**Fig. 3:**
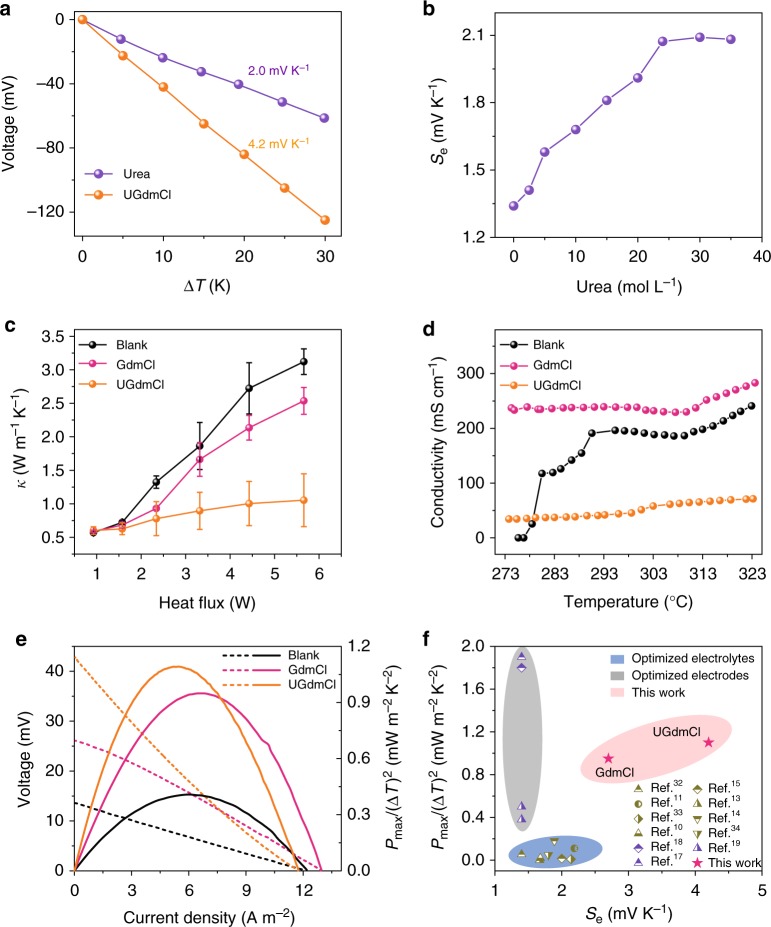
Performance of the optimized TGC systems. **a** The Seebeck effects of the [Fe(CN)_6_]^3−^/[Fe(CN)_6_]^4−^ electrolyte containing only urea or urea/GdmCl composite (UGdmCl). The concentrations of urea and GdmCl were 24 and 2.6 M, respectively. **b** Dependence of the Seebeck coefficient on the concentration of urea. **c** Dependence of the thermal conductivity on the input heat flux. The error bar is received from the temperature gradient at three different positions of the cross-section of cells according to the regarding method in Supplementary Note 4. **d** Dependence of the electrical conductivity on the temperature. **e** Voltage output and the corresponding power output. The temperature difference was 10 K (“cold” side at 293 K, “hot” side at 303 K). **f** Comparison of the *P*_max_/(Δ*T*)^2^ and *S*_e_ values with those reported in the literature

In addition to strong chaotropic cations, we found that highly soluble amide derivatives enhance the Seebeck effect of the [Fe(CN)_6_]^3−^/[Fe(CN)_6_]^4−^ aqueous electrolyte. Urea is a low-cost amide species that can strongly impact the hydrogen-bonding network and hydration shell of ions in water^28,29^. A high *S*_e_ value of ~2.0 mV K^−1^ is achieved solely by adding urea to the pristine electrolyte (Fig. 3a). Note that the *S*_e_ value increases with increasing urea concentration and reaches the maximum when oversaturated urea with a concentration of ~24 M is added (Fig. 3b) In addition, we evaluate the influence of other six amide derivatives on the Seebeck effect of the [Fe(CN)_6_]^3−^/[Fe(CN)_6_]^4−^ aqueous electrolyte (Supplementary Table 1 and Supplementary Note 3). The results show that highly soluble amide species (acrylamide, propanamide, and formamide) enhance the Seebeck effect, whereas poorly soluble amide species (thiourea, biuret, and hydroxycarbamide) have little effect. The results confirm that a high concentration of amide derivatives is essential for enhancing the Seebeck effect.

Interestingly, an extremely high *S*_e_ value of ~4.2 mV K^−1^ is achieved when adding optimized urea (24 M) and GdmCl (2.6 M) simultaneously (labeled as UGdmCl in Fig. 3a, Supplementary Figure 6). This high *S*_e_ value far exceeds previously reported values for aqueous and organic electrolytes^16^ and is an order of magnitude higher than those of state-of-the-art rigid and flexible thermoelectric materials, such as Bi_2_Te_3_ (~0.2 mV K^−1^)^30^ and poly(3,4-ethylenedioxythiophene) (~0.16 mV K^−1^)^31^, under ambient conditions. Urea and GdmCl appear to have synergistic effects on enhancing *S*_e_ of [Fe(CN)_6_]^3−^/[Fe(CN)_6_]^4−^ electrolytes. However, we do not observe a synergistic effect in the composites of GdmCl and other highly soluble amide species (Supplementary Figure 7). The MD simulation indicates that the polar urea molecules form stronger hydrogen bonds with [Fe(CN)_6_]^3−^ than with [Fe(CN)_6_]^4−^ (Supplementary Figures 8, 9 and Supplementary Note 1). Since urea prefers to bond with [Fe(CN)_6_]^3−^ while GdmCl prefers to bond with [Fe(CN)_6_]^4−^, the entropy difference between the redox couple is dramatically increased in the composite of urea and GdmCl, resulting in a synergistic effect (Supplementary Figure 10).

The performances of TGC systems can be evaluated by the figure of merit (ZT)

2 $${\mathrm{ZT}} = {S_{\mathrm{e}}^2}\sigma T/\kappa,$$

where *T* is the absolute temperature, *σ* is the electrical conductivity, and *κ* is the thermal conductivity. According to Eq. 2, in addition to *S*_e_, the thermal conductivity (*κ*) and ionic conductivity (*σ*) are also important features of TGCs. Figure 3c shows the trends of *κ* for three TGC systems measured by the steady-state method (Supplementary Figure 11 and Supplementary Note 4). For the blank and GdmCl systems, *κ* significantly increases with increasing heat input due to the intense heat convection at a large temperature difference. By contrast, *κ* increases slightly for the UGdmCl system because heat convection is dramatically suppressed by the oversaturated urea, resulting from an increase in the viscosity of the electrolyte. Thus, a larger temperature difference is created in the UGdmCl system than in the blank and GdmCl systems at the same heat input (Supplementary Figure 12). In general, thermal conductivity and ionic conductivity have a trade-off relationship and are difficult to optimize simultaneously. Compared with the blank and GdmCl systems, the UGdmCl system has the lowest conductivity due to slow mass transport while still remaining at a high level: approximately 50 mS cm^−1^ at room temperature (Fig. 3d). Note that the ionic conductivity of the blank system significantly decreases below 278 K because of the crystallization of the redox ions. However, the ionic conductivities of GdmCl and UGdmCl confer stability, indicating that urea and guanidinium can inhibit crystallization of the redox ions at low temperature. Moreover, the performance of blank system decays, while the performance of GdmCl and UGdmCl remain excellent at cold temperature (Supplementary Figure 13 and Supplementary Note 5). The optimized TGC systems therefore can be stably operated in cold environments. Figure 3e shows the power outputs of TGC systems under temperature difference of 10 K. *V*_oc_ is significantly higher for the optimized systems than for the blank system, whereas *I*_sc_ does not increase significantly due to the slow mass transport. From the *I*–*V* curves, *P*_max_/(Δ*T*)^2^ is obtained, with values of 0.41, 0.95, and 1.10 mW K^−2^ m^−2^ for blank, GdmCl, and UGdmCl, respectively. The *P*_max_/(Δ*T*)^2^ value for the UGdmCl system is enhanced by nearly threefold compared to the blank system due to the synergistic effect of urea and guanidine hydrochloride.

Ideally, a TGC system with a high *S*_e_ and *P*_max_/(Δ*T*)^2^ value is crucial for efficiently producing available electrical energy from a small temperature difference. Here, we compare the *S*_e_ and *P*_max_/(Δ*T*)^2^ values of our systems with those reported in the literature for typical planar and static TGC systems, as shown in Fig. 3f and Supplementary Table 2. Baughman and colleagues^17–19^ applied optimized carbon-based electrodes to produce high *P*_max_/(Δ*T*)^2^ values of approximately 0.38−1.9 mW K^−2^ m^−2^ but did not enhance the *S*_e_ value. By contrast, other research groups^9,10,12–14,32–34^ have focused on enhancing *S*_e_ in optimized electrolytes, yielding moderately high *S*_e_ values of approximately 1.4–2.2 mV K^−1^ but inferior *P*_max_/(Δ*T*)^2^ values of <0.3 mW K^−2^ m^−2^. By contrast, our optimized TGC system combines the highest *S*_e_ value of 4.2 mV K^−1^ and a high *P*_max_/(Δ*T*)^2^ value of 1.1 mW K^−2^ m^−2^:

The energy conversion efficiency (*η*_r_) relative to the Carnot efficiency limit of a heat engine is as follows (Eq. 3):

3 $$\eta _{\mathrm{r}} = \frac{\eta }{{\left( {{\mathrm{\Delta }}T/T_{\mathrm{H}}} \right)}} = \frac{{P_{\mathrm{max}}/\left( {\kappa \cdot \frac{{\Delta {{T}}}}{d}} \right)}}{{\left( {{\mathrm{\Delta }}T/T_{\mathrm{H}}} \right)}} = \frac{{\left( {P_{{\mathrm{max}}}/({\mathrm{\Delta }}T)^2} \right)d \cdot T_{\mathrm{H}}}}{\kappa },$$

where the electrode separation distance (*d*) is 10 mm, the hot-side temperature (*T*_H_) is 303 K, and *κ* is the thermal conductivity at a Δ*T* of 10 K (Supplementary Figure 14). Due to the high power density and low thermal conductivity, the *η*_r_ value of 0.79% for the UGdmCl electrolyte is more than fivefold higher than that of the blank system (Supplementary Figure 15).

### The performances of a prototype module

**Fig. 4:**
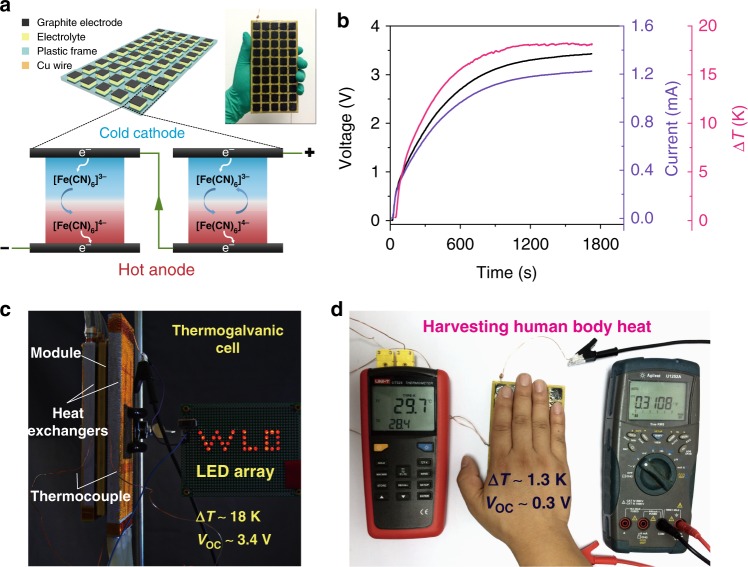
Demonstration of the harvest of low-grade heat by a prototype module. **a** Schematic of the structure and a photograph of the module. **b** Voltage and current curves of the module corresponding to the applied temperature difference. **c** Image of the module directly powering an LED array containing 29 red LED lamp beads. The module was sandwiched by two aluminum heat exchangers with water as the heat transfer fluid. The temperatures of the cold and hot water were controlled at approximately 278 and 333 K, respectively. Because of inevitable thermal conduction in the module and thermal contact resistance between the module and heat exchangers, the Δ*T* of 18 K built on the module at the steady state was smaller than that of the two heat exchangers. **d** Harvesting heat energy from the human body. The module was placed on a steel platform as a heat sink with a temperature of approximately 298 K and then covered by one hand. After several minutes, a stable temperature difference of 1.3 K was built, which induced an output of approximately 0.3 V by the module. The temperature and voltage were measured by a thermometer (left) and multimeter (right), respectively

To demonstrate the potential applications of our optimized systems for harvesting low-grade thermal energy, we fabricated a prototype module containing 50 UGdmCl units (1 cm^2^ area, 0.5 cm thickness) connected by Cu wires in series, as illustrated in Fig. 4a. The device generates *V*_oc_ and *I*_sc_ values of 3.4 V and 1.2 mA, respectively, at an applied Δ*T* of 18 K (Fig. 4b) and can directly power an LED array (Fig. 4c and Supplementary Movie 1). Note that the average *S*_e_ value for the module is calculated to be 3.8 mV K^−1^, which is lower than the value of 4.2 mV K^−1^ because of the inevitable thermal contact resistance between the module and heat sources. Furthermore, the module is robust (Supplementary Movie 2) that can also harvest heat from the human body, and a stable voltage of more than 0.3 V is generated by a small temperature difference of approximately 1.3 K, as shown in Fig. 4d. In addition, our module can harvest waste heat in a cold environment (Supplementary Figure 16a) or from a refrigerator (Supplementary Figure 16b).

## Discussion

A low-cost TGC system combining the highest Seebeck coefficient and a high power density was developed by introducing urea and guanidinium into an aqueous electrolyte containing 0.4 M [Fe(CN)_6_]^3−^/[Fe(CN)_6_]^4−^. The underlying mechanism of the enhancement of the Seebeck coefficient is the significant increase in the entropy difference of the redox couple due to the synergistic interactions between urea/guanidinium and the redox couple. Guanidinium is prone to bond with [Fe(CN)_6_]^4−^ rather than [Fe(CN)_6_]^3−^ based on the ion specificity, whereas urea has a stronger affinity for [Fe(CN)_6_]^3−^ than for [Fe(CN)_6_]^4-^. These differences in affinity synergistically enlarge the entropy difference of the redox couple, thereby significantly increasing the Seebeck effect. The performance of the electrolyte system was demonstrated with a module that integrated 50 optimized cells and generated usable electrical energy from several low-temperature heat sources, indicating the promising potential of this system for efficiently harvesting low-grade thermal energy. Further improvements in the performance of the device might be achieved by using highly conductive and porous electrode materials^17^, optimizing the trade-off between ionic conductivity and thermal conductivity by applying a porous thermal separator^17^. In addition, a flexible and wearable TGC could be designed by using gel electrolytes for harvesting body heat^35,36^.

## Methods

### Materials

All chemical reagents were purchased from Sigma-Aldrich Trading Co. Ltd. and used without further purification. Graphite electrodes with an electrical resistivity of approximately 10 µΩ m were commercial products purchased from Graphite Material Company Ltd. Water obtained from a Milli-Q system (Simplicity, Millipore, France) was used in all experiments.

### Performance characterization of the TGC

The performance of the TGC was measured by the typical planar cell device shown in Supplementary Figure 1. The open-circuit voltage (*V*_oc_) of the cells was measured with a Keithley 2000 multimeter, and the corresponding temperature difference was recorded by a thermocouple data logger (USB-TC-08, Pico Technology, St. Neots, UK). The current–voltage characterization of the device was performed with a Keithley 2400 instrument. There are approximately100 points between 0 V to open-circuit voltage. The voltage sweep rate is 0.1 s per point. The thermal conductivities of the TGCs were measured by the steady-state method (Supplementary Figure 11 and Supplementary Note 4). The ionic conductivities of the TGCs were measured with a conductivity meter (Mettler Toledo S-230).

### Mechanism characterization

The interactions between GdmCl and [Fe(CN)_6_]^4−^/[Fe(CN)_6_]^3−^ were characterized with a UV–Vis spectrophotometer (LabRAM HR800) and an X–ray photoelectron spectrometer (ESCALab250). Because the concentration of 0.4 M [Fe(CN)_6_]^4−^/[Fe(CN)_6_]^3−^ was too high for UV–Vis measurements, we diluted the solution to a concentration of 0.2 mM for UV–Vis measurements. The solid powders of GdmCl, K_3_[Fe(CN)_6_] and K_4_[Fe(CN)_6_] were dried in a vacuum oven at 333 K for 24 h before the XPS measurements. The samples of GdmCl/K_3_[Fe(CN)_6_] and GdmCl, K_4_[Fe(CN)_6_] were prepared for the XPS measurements by drying their composite solutions in a vacuum oven at 333 K for 48 h. The MD simulations were performed using the MD software package Gromacs 4.6 in the NPT ensemble at 298 K and 1 atm (detailed information is provided in Supplementary Note 1).

### Module preparation

The module containing 50 integrated UGdmCl units consisted of a polyamide frame (commercial sources), graphite electrodes, electrolytes and copper (Cu) wires. The polyamide frame had a size of 160 × 80 × 8 mm^3^ and contained 50 cells (10 × 10 × 5 mm^3^). Fifty graphite electrodes (12 × 12 × 2 mm^3^) were first fixed to one side of the polyamide frame with epoxy glue. Then, the cells were filled with the electrolyte. Finally, all cells were sealed with an additional 50 graphite electrodes. The cells were connected in series by Cu wires. To prevent the leakage, the whole module was sealed by epoxy resin glue. As shown in Fig. 4a, the module was sandwiched between two aluminum (Al) heat exchangers. The temperature difference across the module was created by pumping circulating hot water (333 K) and cold water (278 K) in the two heat exchangers. The *V*_oc_ of the cells was measured with a Keithley 2000 multimeter, and the corresponding temperature difference was recorded by a thermocouple data logger. The current was measured with a Keithley 2400 instrument. The LED array consisted of 29 red LED lamp beads in parallel.

## Electronic supplementary material

Supplementary Information

Description of Additional Supplementary Files

Supplementary Movie 1

Supplementary Movie 2

## Data Availability

The data that support the findings of this study are available from the corresponding author upon reasonable request.
