# Supplementary material for: Aqueous thermogalvanic cells with a high Seebeck coefficient for low-grade heat harvest
Source: Nat Commun. 2018 Dec 4;9:5146. doi: 10.1038/s41467-018-07625-9 (PMC6279834; doi:10.1038/s41467-018-07625-9)
Supplement: Supplementary file 1 — Supplementary Information [file 41467_2018_7625_MOESM1_ESM.pdf]

Aqueous thermogalvanic cells with a high Seebeck coefficient for low-grade heat harvest

(Supplementary Information)

Duan et al.

Table of contents:

Supplementary Figures 2
Supplementary Tables 11
Supplementary Notes 13
Supplementary References 19

Supplementary Figures

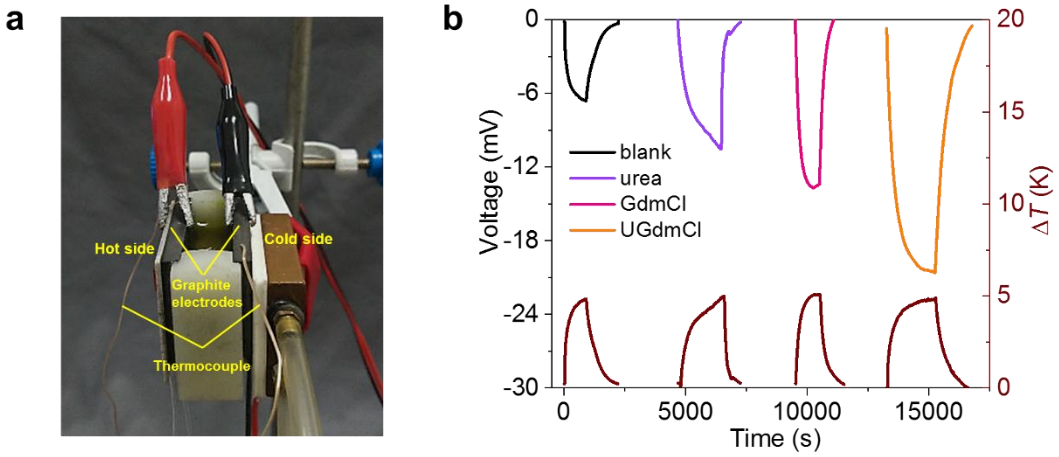

Supplementary Figure 1| Test of the Seebeck effect by a planar device. a, Photograph of the planar device. The device consisted of two graphite electrodes and a polyamide frame. The distance between the two electrodes was 15 mm. The hot side was heated by a metal ceramic heater (MCH). The cold side was cooled by a thermoelectric cooler contacting a water-cooling plate. The thermocouple wires were inserted into holes on the top of the graphite electrodes. **b,** The instantaneous voltage for four TGC systems at temperature differences of 0 to 5 K. At a ΔT of 5 K, the temperatures at the cold side and the hot side were ca. 298 and 303 K.

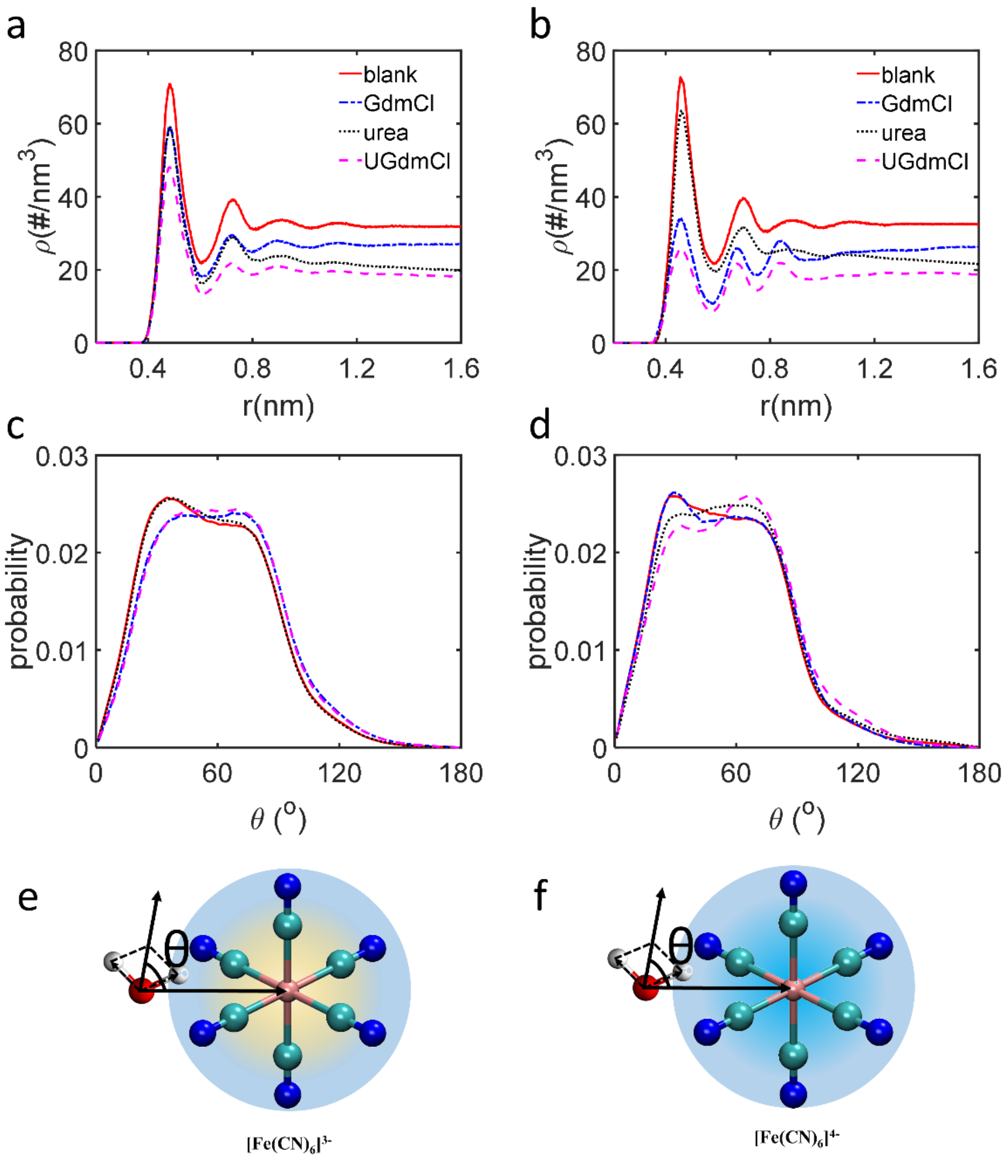

Supplementary Figure 2| Radial density profiles and orientation profiles. Radial density profiles of the $[Fe(CN)_6]^{3-}$@water (**a**) and $[Fe(CN)_6]^{4-}$@water (**b**) in different media. Orientation profiles of water around $[Fe(CN)_6]^{3-}$ (**c, e**) and $[Fe(CN)_6]^{4-}$ (**d, f**) in different media. θ is the angle between the dipole of water and the direction of O (in water) to Fe (in $[Fe(CN)_6]^{3-}$ or $[Fe(CN)_6]^{3-}$ species).

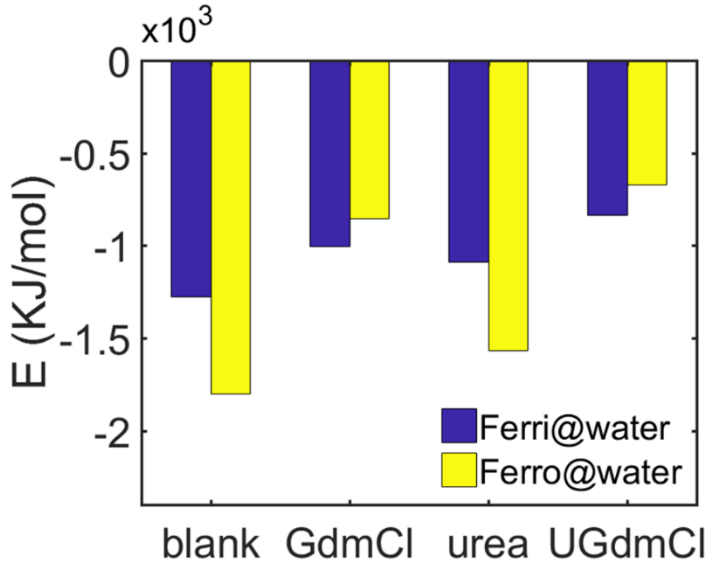

Supplementary Figure 3| The interaction energy between the anion and water. The interaction energy of $[Fe(CN)_6]^{3-}$/$[Fe(CN)_6]^{4-}$ and water in the first solvation shell as a function of different media.

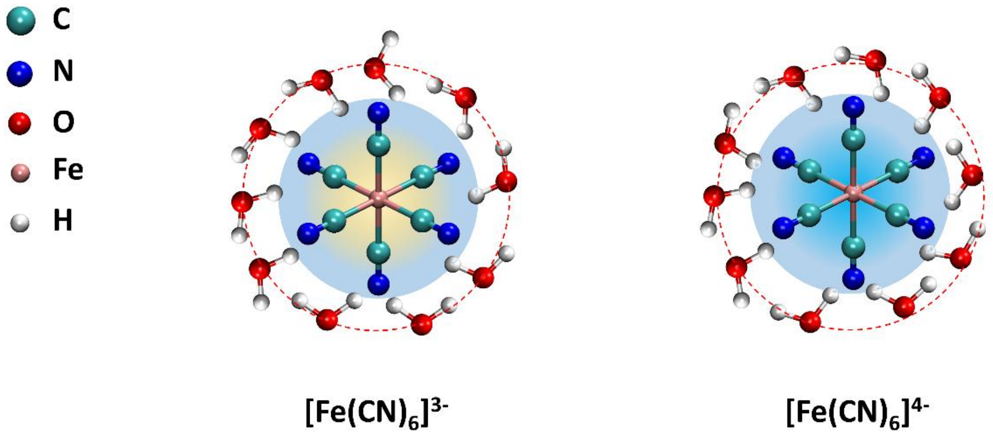

Supplementary Figure 4| Schematic solvation shells in pristine water. The schematic solvation shells of $[Fe(CN)_6]^{3-}$/$[Fe(CN)_6]^{4-}$ are plotted according to the molecular dynamics (MD) simulations in the pristine solution.

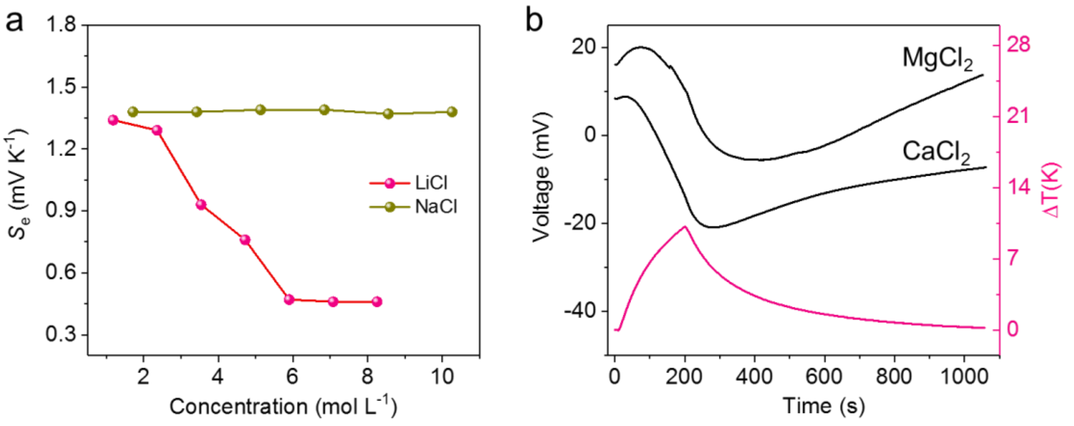

Supplementary Figure 5| The effects of kosmotropic species on S_e. a, The dependence of the S_e value on the concentration of LiCl and NaCl. **b**, The instantaneous voltage and corresponding temperature difference for the $[Fe(CN)_6]^{3-}/[Fe(CN)_6]^{4-}$ electrolyte containing $MgCl_2$ or $CaCl_2$. At a ΔT of 10 K, the temperatures on the cold side and the hot side were ca. 293 and 303 K, respectively. The concentrations of $MgCl_2$ and $CaCl_2$ were both 3 mol L^{-1}.

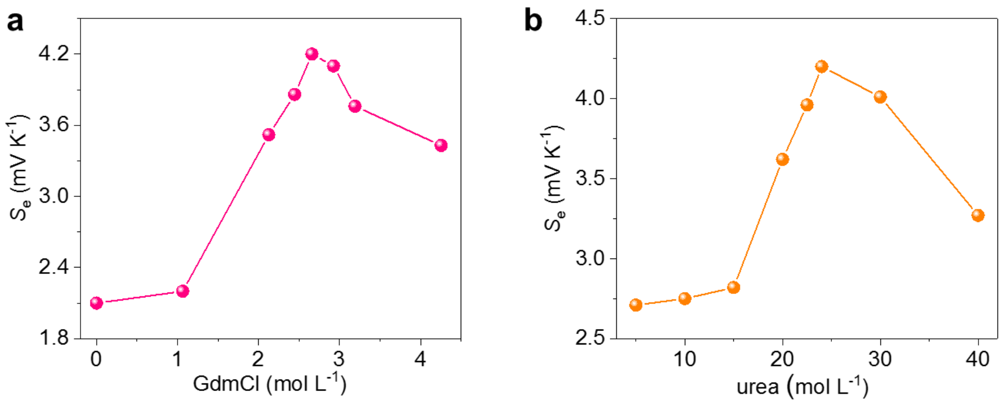

Supplementary Figure 6| Synergistic enhancement effects. a, Dependence of S_e of the TGC on the concentration of GdmCl. For the experiment, urea at an optimized concentration of 24 mol L^{-1} was added to the electrolytes. **b**, Dependence of S_e of the TGC on the concentration of urea in the electrolyte that contains GdmCl with an optimized concentration of 2.6 mol L^{-1}.

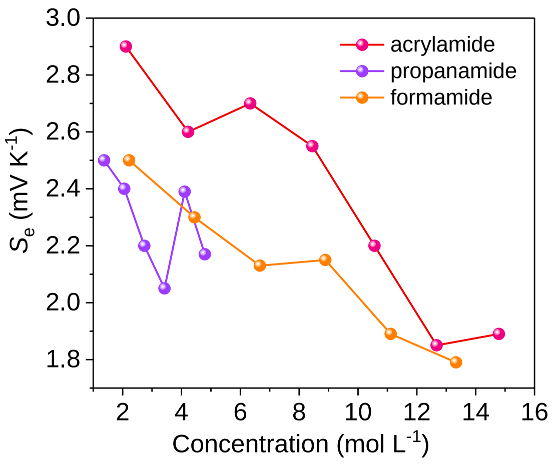

Supplementary Figure 7| The enhancement effects of amide species. Dependence of the Seebeck coefficient of the TGC on the concentrations of different highly soluble amide species. For the experiment, GdmCl at an optimized concentration of 2.6 mol L^{-1} was added to the electrolytes.

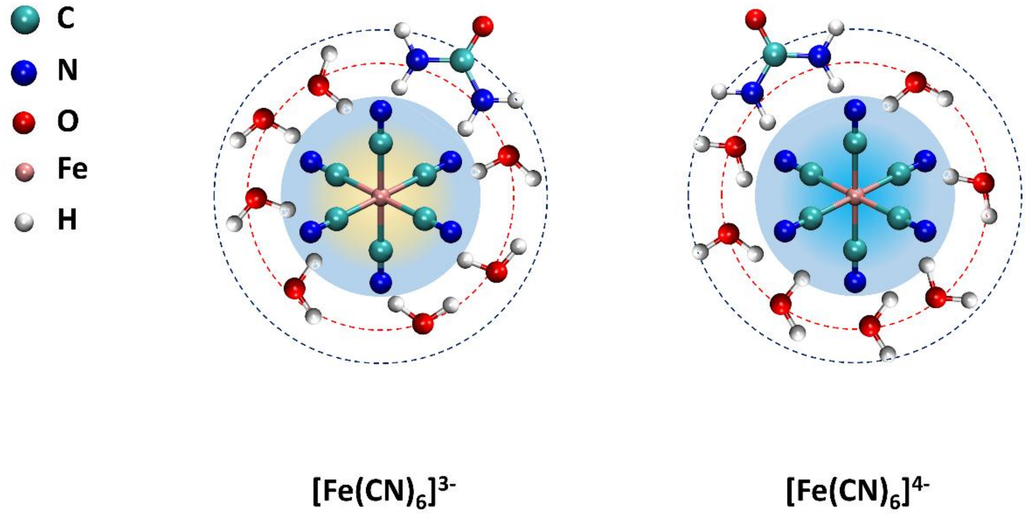

Supplementary Figure 8| Schematic solvation shells in the urea system. The schematic solvation shells of $[Fe(CN)_6]^{3-}$/$[Fe(CN)_6]^{4-}$ are plotted according to the molecular dynamics (MD) simulations in the solution containing optimized concentration of urea.

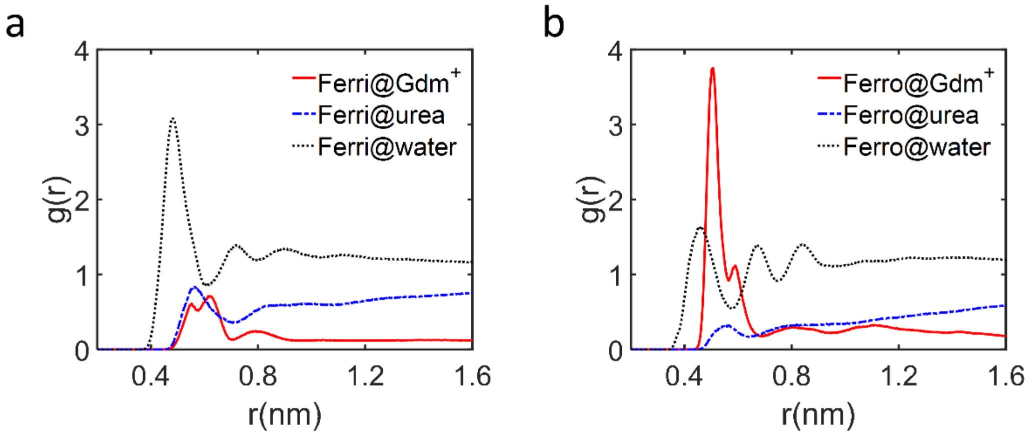

Supplementary Figure 9| The RDF g(r) for ferri/ferrocyanide in different media. The RDF g(r) for different systems: $[Fe(CN)_6]^{3-}$ with Gdm^+ (red line in **a**);$[Fe(CN)_6]^{3-}$ with urea (blue dot-dash line in **a**); $[Fe(CN)_6]^{3-}$ with water (black dash line in **a**); $[Fe(CN)_6]^{4-}$ with Gdm^+ (red line in **b**); $[Fe(CN)_6]^{4-}$ with urea (blue dot-dash line in **b**); $[Fe(CN)_6]^{4-}$ with water (black dash line in **b**). To show convenience, the RDFs g(r) for $[Fe(CN)_6]^{3-}$ with Gdm^+ (and $[Fe(CN)_6]^{4-}$ with Gdm^+) have been decreased by 10 times.

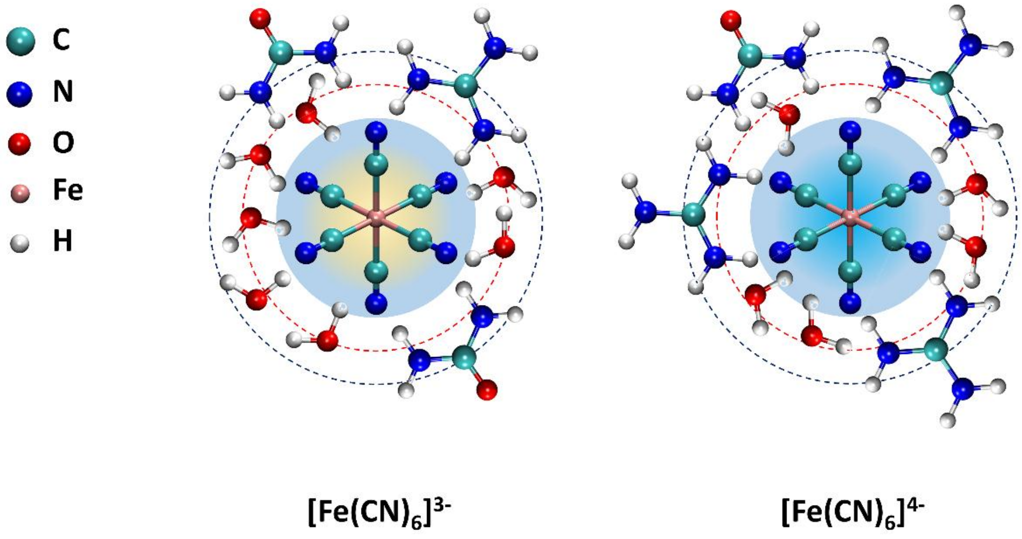

Supplementary Figure 10| Schematic solvation shells in the UGdmCl system. The schematic solvation shells of $[Fe(CN)_6]^{3-}$/$[Fe(CN)_6]^{4-}$ are plotted according to the molecular dynamics (MD) simulations in the solution containing both optimized concentrations of GdmCl and urea.

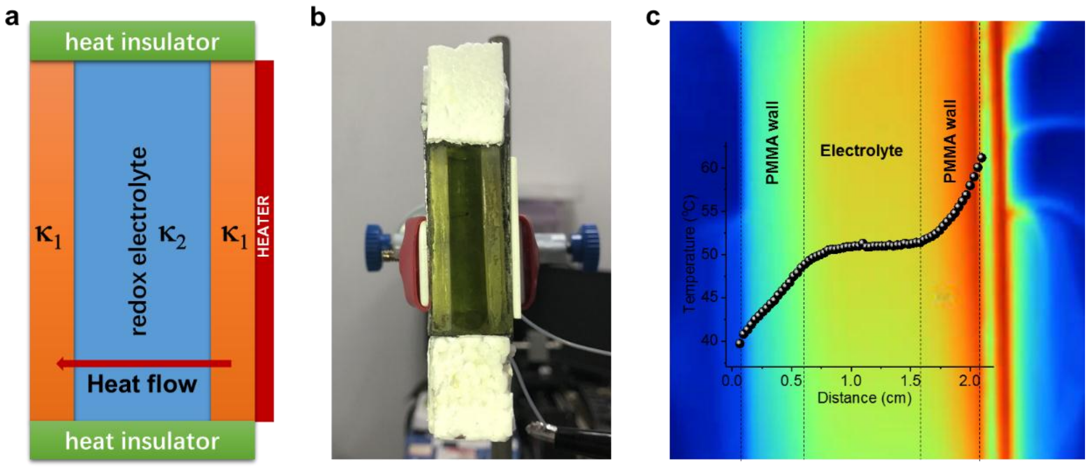

Supplementary Figure 11| Thermal conductivity measurement. a–b, Schematic and photograph of the cell used for the thermal conductivity measurement. **c**, The infrared thermography and corresponding temperature gradient across the box were measured by an infrared imaging device (TiX520, Fluke, USA).

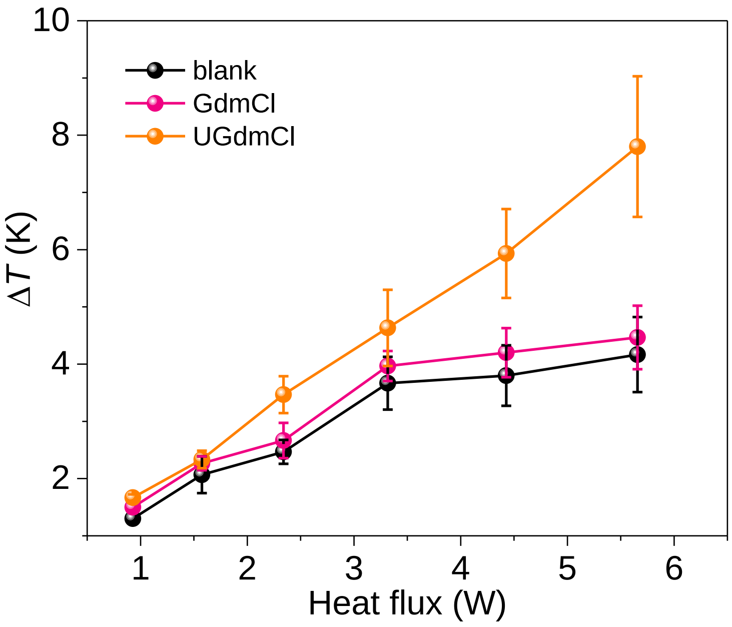

Supplementary Figure 12| The temperature differences in the different systems. Dependence of the temperature differences across the TGC on heat flux. The temperature difference increased with increasing heat input. For the urea and GdmCl systems, the increase in the temperature difference slowed at high heat input due to extensive heat convection. However, the temperature difference for the UGdmCl systems linearly increased with the heat input, further indicating suppression of convection. The error bar is received by measuring temperature gradient at three different positions on the cross-section of cells by an infrared imaging device as shown in Supplementary Figure 11c.

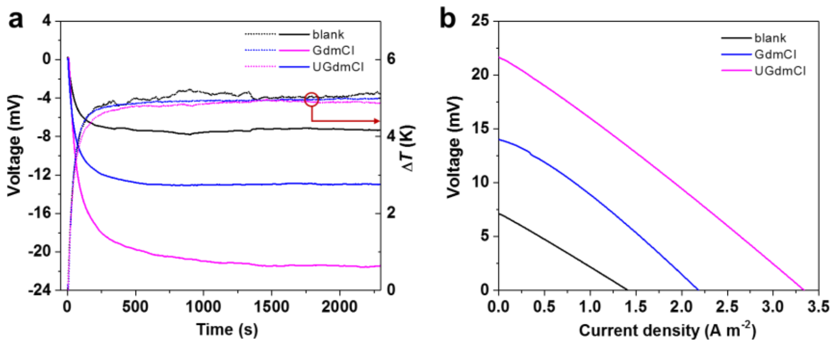

Supplementary Figure 13| The performances of TGC at low temperature. a, The dependence of open-circuit voltage and corresponding temperature difference on time. The cold side and hot side were controlled at approximately 273 K and 279 K, respectively. **b,** The steady-state current-voltage curves.

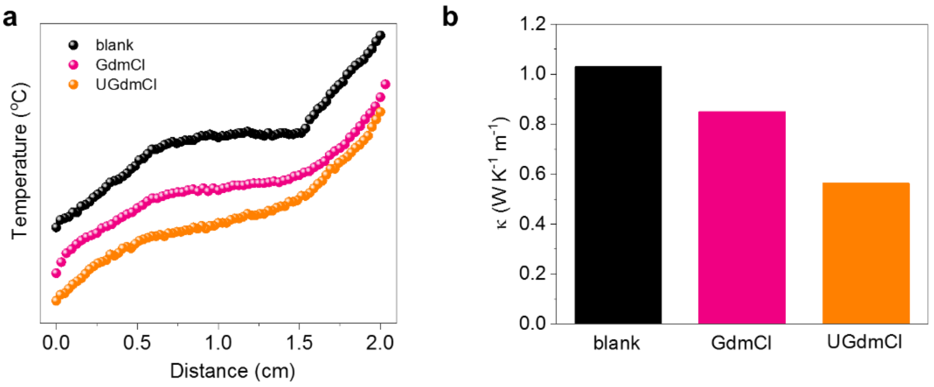

Supplementary Figure 14| Thermal conductivities of the different TGC systems. a, Temperature gradient across the cell. The temperatures at the cold and hot sides were 293 and 303 K, respectively. **b,** Thermal conductivities of the different TGC systems calculated from the results in the left according to the Supplementary Equation 2 in Supplementary Note 4. Compared with the blank and GdmCl systems, the UGdmCl system containing a high concentration of urea had higher viscosity in the electrolyte, significantly suppressing the heat convection and leading to the lowest thermal conductivity in the UGdmCl system.

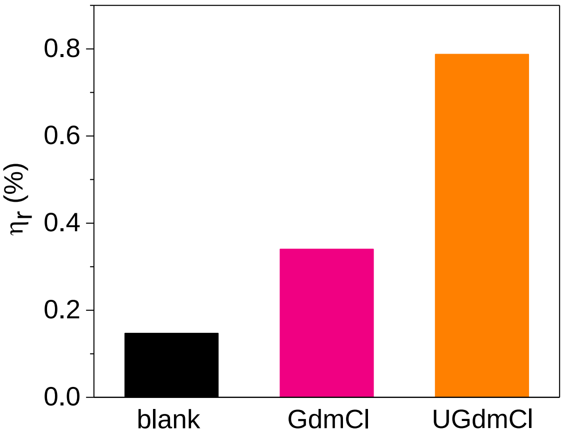

Supplementary Figure 15| Efficiency of our TGC systems. Thermal–electrical conversion efficiency relative to the Carnot cycle (η_r) for different TGC systems. η_r is calculated according to Equation 3 in the main text.

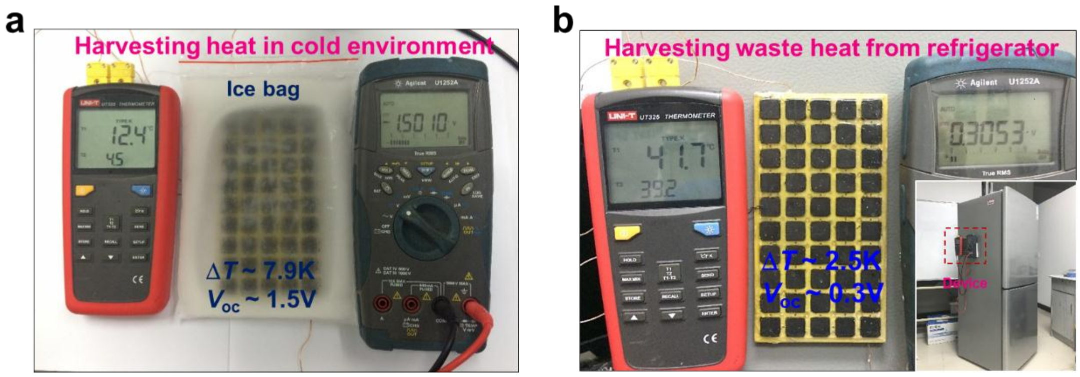

Supplementary Figure 16| Demonstration of the harvest of low-grade heat energy. **a**, Harvesting heat in a cold environment. To illustrate the operation at cold temperature, the top side of the module was covered with an ice bag (~273 K), and its other side was contacted with a steel platform. At the steady state, a voltage of ~1.5 V was achieved at a temperature difference of ~7.9 K. The average S_e value (S_e=1.5/50/7.9) for the module was calculated to be 3.8 mV K^{-1}. **b**, Harvesting heat from a refrigerator. At the steady state, a voltage of ~0.3 V was achieved at a temperature difference of ~2.5 K. The average S_e value (S_e=0.3/50/2.5) for the module was calculated to be 2.4 mV K^{-1}. The S_e value was lower than the value of 4.2 mV K^{-1} for UGdmCl because of the inevitable thermal contact resistance between the module and heat sources.

Supplementary Tables

Supplementary Table 1| The Seebeck effect containing amide derivatives. The table presents the highest Seebeck coefficient and corresponding concentration of the different amide derivatives.

Amide derivative	Solubility	Optimized concentration (mol L^{-1})	Seebeck coefficient (mV K^{-1})
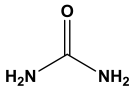 **urea**	highly soluble	24.0	2.0
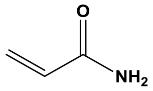 **acrylamide**	highly soluble	10.6	3.0
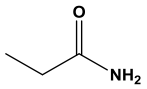 **propanamide**	highly soluble	3.4	2.6
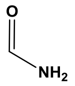 **formamide**	highly soluble	8.9	1.7
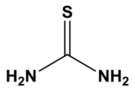 **thiourea**	slightly soluble	1.3	1.5
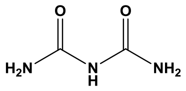 **biuret**	slightly soluble	0.2	1.5
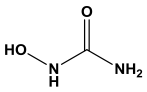 **hydroxycarbamide**	slightly soluble	1.5	1.4

Supplementary Table 2 | Performance comparison. Comparison of the S_e and $P_{max}/(\Delta T)^2$ values of our TGC systems with those reported in the literatures regarding electrode materials optimization and the enhancement of S_e in optimized electrolytes.

Redox	Electrolyte	Electrode	S_e（mV K^{-1}）	$P_{max}/(\Delta T)^2$ (mW m^{-2} K^{-2})	[d]Reference
Li^+/Li	Ionic liquid	Lithium	1.4	0.054	Ref.32
CoII/III$(bpy)_3(NTf_2)_{2/3}$	Ionic liquid	Platinum	2.19	0.11	Ref.11
[a]Fc/I_3^-	Ionic liquid	Platinum	1.67	0.0000001	Ref.13
[b]CoII/III$(bpy)_3(NTf_2)_{2/3}$	Ionic liquid	Platinum	2.14	0.0089	Ref.33
CoII/III$(bpy)_3(NTf_2)_{2/3}$	Ionic liquid	Platinum	1.89	0.18	Ref.14
CoII/III$(bpy)_3(NTf_2)_{2/3}$	Ionic liquid	Platinum	1.68	0.019	Ref.10
CoII/III$(bpy)_3(NTf_2)_{2/3}$	Ionic liquid	Platinum	1.8	0.049	Ref.34
[c]**α**-CD-I^-/I_3^-	Aqueous	Platinum	2.0	0.015	Ref.15
$Fe(CN)_6^{4-}/Fe(CN)_6^{3-}$	Aqueous	Carbon nanotube	1.4	0.38, 0.5	Ref.19
$Fe(CN)_6^{4-}/Fe(CN)_6^{3-}$	Aqueous	Carbon nanotube	1.4	1.8	Ref.18
$Fe(CN)_6^{4-}/Fe(CN)_6^{3-}$	Aqueous	Activated carbon cloth	1.4	1.9	Ref.17
$Fe(CN)_6^{4-}/Fe(CN)_6^{3-}$	Aqueous	Graphite	2.7	0.95	This work
$Fe(CN)_6^{4-}/Fe(CN)_6^{3-}$	Aqueous	Graphite	4.2	1.1	This work

[a]Fc refers to ferrocene.

[b]CoII/III$(bpy)_3(NTf_2)_{2/3}$ refers to a type of cobalt(II/III) tris(bipyridyl) redox couple.

[c]**α**-CD refers to **α**-cyclodextrin.

[d]The references cited in the main text.

Supplementary Notes

Supplementary Note 1

Molecular dynamics (MD) simulations: The molecular dynamics (MD) simulations were performed using the MD software package Gromacs 4.6[1] in the NPT ensemble at 298 K and 1 atm. Ferricyanide ($[Fe(CN)_6]^{3-}$) and ferrocyanide ($[Fe(CN)_6]^{4-}$) anions were modelled by all-atom force fields developed by Giacomo *et al*[2]. The force field parameters were taken from ref. 3 for K^+ ions. The SPC/Fw model[4], which is capable of taking into account water flexibility[2], was adopted for water. Furthermore, the force field parameters for urea and GdmCl were based on the OPLS model[5,6]. The detailed simulation setup with the number of ions/molecules is summarized in Table 1 for all studied simulation systems.

Periodic boundary conditions were used in all three directions of the MD system. A cut-off distance of 1.4 nm was employed for both electrostatic interactions and van der Waals terms, whereas the long-range electrostatic interactions were accounted for through the particle mesh Ewald (PME) method[7]. Temperature and pressure were controlled through the Berendsen[8] weak-coupling scheme with coupling constants of 0.1 and 1 ps, respectively. The leapfrog integration algorithm was used to solve the equations of motion with a time step of 0.5 fs. The trajectory was saved every 0.5 ps. Each simulation was equilibrated within 10 ns. Another 20-ns production run was subsequently performed for analysis.

The radial density profiles between the centres of mass of the ion and water molecules when adding different amounts of GdmCl, urea and UGdmCl are shown in **Supplementary Figure 2a** ($[Fe(CN)_6]^{3-}$@SOL) and **Supplementary Figure 2b** ($[Fe(CN)_6]^{4-}$@SOL). The orientation profiles of water around ferri or ferro species are shown in **Supplementary Figure 2c** ($[Fe(CN)_6]^{3-}$@SOL) and **Supplementary Figure 2d** ($[Fe(CN)_6]^{4-}$@SOL). Before adding GdmCl and/or urea, water molecules in 0.4 M $K_3[Fe(CN)_6]$ solution are farther from $[Fe(CN)_6]^{3-}$, with a peak position at approximately 4.8 Å, whereas the peak of the radial density profile for $[Fe(CN)_6]^{4-}$ is approximately 4.6 Å in 0.4 M $K_4[Fe(CN)_6]$ solution. The higher charge of $[Fe(CN)_6]^{4-}$ results in a more closely "packed" hydration shell, similar to previous work[2] and ultrafast spectroscopy results[9]. The schematic solvation structures of $[Fe(CN)_6]^{3-}$/$[Fe(CN)_6]^{4-}$ in pristine solution are shown in **Supplementary Figure 4**. Upon the addition of GdmCl or urea, the hydration capacities of both $[Fe(CN)_6]^{3-}$ and $[Fe(CN)_6]^{4-}$ decrease to a certain degree. Although the size of the first solvation shell does not change greatly, some water molecules in the first solvation shell are squeezed out. To provide a quantitative description, the hydration energy of the first solvation energy is calculated[10] (**Supplementary Figure 3**). The hydration energy of the ferri species decreases from -1276.48 kJ mol^{-1} to -1007.25 kJ mol^{-1}, -1091.18 kJ mol^{-1} and -837.71 kJ mol^{-1} when adding GdmCl, urea and urea/GdmCl, respectively. For the ferro species, the hydration energy decreases from -1804.42 kJ mol^{-1} to -853.21 kJ mol^{-1}, -1568.97 kJ mol^{-1} and -671.86 kJ mol^{-1}, respectively.

A deep insight into the decrease in the hydration capacity of the $[Fe(CN)_6]^{3-}$ and

$[Fe(CN)_6]^{4-}$ ions can be obtained by examining the interactions between these two anions and other species, as well as the pair correlation function between these anions and water (**Supplementary Figure 2a-b**). When GdmCl salt is added, due to its higher charge, Gdm^+ has a stronger Coulomb interaction with the $[Fe(CN)_6]^{4-}$ complex (-9161.82 kJ mol^{-1}) than with $[Fe(CN)_6]^{3-}$ (-2344.95 kJ mol^{-1}). Thus, more Gdm^+ ions are bound to ferro species, resulting in greater destruction of the water structure. The changes in the schematic solvation structures of $[Fe(CN)_6]^{3-}$/$[Fe(CN)_6]^{4-}$ in GdmCl solution are illustrated in **Figure 2e**. Furthermore, the neutral but polar urea molecules have a stronger attraction to $[Fe(CN)_6]^{3-}$ than to $[Fe(CN)_6]^{4-}$, i.e., the interaction energy between $[Fe(CN)_6]^{3-}$ and urea is -222.86 kJ mol^{-1} within the first shell but -67.60 kJ mol^{-1} for $[Fe(CN)_6]^{4-}$. This difference could be attributed to hydrogen bonds (HBs) formed with anions; that is, there are more HBs for ferri species@urea (2.78 HBs/per molecule) than for the ferro species (1.00 HBs/per molecule). Therefore, the solvation shell (**Supplementary Figure 8**) is disrupted more extensively for the ferri species, and thus the water number density at the first peak of $[Fe(CN)_6]^{3-}$ decreases from 70.89 # nm^{-3} to 59.19 # nm^{-3}; by contrast, the water number density decreases from 72.72 # nm^{-3} to 63.64 # nm^{-3} for the ferro species (**Supplementary Figure 2a-b**).

The schematic solvation structures of $[Fe(CN)_6]^{3-}$/$[Fe(CN)_6]^{4-}$ in the UGdmCl system are shown in **Supplementary Figure 10**. The water molecules pack the first solvation shell and then compactly interact with urea molecules and Gdm^+ cations. However, there are some differences in the solvation shell between the ferri and ferro species. The value of the first peak of urea with the ferri species is slightly larger than with the ferro species. Nevertheless, Gdm^+ has a sharp peak with the ferro species that is 5.3 times higher than that for the ferri species.

To better describe the degree of destruction of the hydration shell, the coordination numbers for water molecules around the two anions are calculated according to the following Equation:

$$N_{AB} = 4\pi \int_0^{R_{\min}} \rho_B g_{AB}(r) r^2 dr \qquad (1)$$

Here, $R_{\min}$ is the first valley of the RDF between the anion and water, and ρ_B is the average number density of the water molecules. As shown in **Supplementary Figure 9**, the coordination number decreases as expected. The hydration shell is destroyed when GdmCl is added; that is, the coordination number of water decreases by 3.50. However, the hydration shell is destroyed more thoroughly for the ferrocyanide anion (its coordination number decreases from 23.58 to 12.03) because of the stronger Coulomb interaction between ferrocyanide and Gdm^+. When urea is added, the ferri species' coordination number is reduced by 4.11, and the coordination number of the ferro species is reduced by 1.96. The damaged solvation shell induced a higher entropy, thus producing a larger Seebeck coefficient.

Supplementary Note 2

The effect of typical kosmotropic cations on S_e: Ion specificities or Hofmeister effects are ubiquitous in biological and chemical systems (Ref.24 in the main text). The Czech scientist Franz Hofmeister first discovered these effects in 1888 during experiments on salt-induced protein precipitation. In general, the ionic hydration strength of ions follows the series SO_4^{2-} > HPO_4^{2-}> CH_3COO^- > F^- > Cl^- > Br^-> NO_3^- > ClO_3^- > I^- > SCN^- > ClO_4^- for anions and Mg^{2+} > Ca^{2+} > Li^+ > Na^+ > K^+ > Rb^+ > Cs^+ > NH_4^+ > $N(CH_3)_4^+$ > guanidinium$^+$ for cations[11]. Ions are usually categorized as chaotropes or kosmotropes based on their perceived influence on the water structure. The ions on the left side of the series, defined as kosmotropes, exhibit strong interactions with water molecules, whereas the ions on the right side of the series, defined as chaotropes, are weakly hydrated by water molecules. In general, the chaotropic (kosmotropic) cations and chaotropic (kosmotropic) anions tend to bond together based on the ion specificity effect, whereas the opposite phenomena are observed between the kosmotropic cation (anion) and the chaotropic anion (cation)[12]. $[Fe(CN)_6]^{3-}/[Fe(CN)_6]^{4-}$ are confirmed to be strong chaotropic anions, although their accurate positions in the Hofmeister series are unclear (Ref.23 in the main text). Kosmotropic cations such as Li^+, Na^+, Mg^{2+} and Ca^{2+} are expected to barely enhance the S_e of the $[Fe(CN)_6]^{3-}/[Fe(CN)_6]^{4-}$ electrolytes. As shown in **Supplementary Figure 5a**, NaCl had little influence on S_e, whereas LiCl had an inhibiting effect on S_e. Furthermore, $MgCl_2$ and $CaCl_2$ eliminated the Seebeck effect of the $[Fe(CN)_6]^{3-}/[Fe(CN)_6]^{4-}$ electrolytes. Their corresponding voltage did not change linearly with the temperature difference (**Supplementary Figure 5b**). We proposed that the moderately kosmotropic Na^+ had little interaction with $[Fe(CN)_6]^{3-}/[Fe(CN)_6]^{4-}$ and thus hardly affected S_e. The strongly kosmotropic cations, including Li^+ Mg^{2+} and Ca^{2+}, which have high charge density, induced the precipitation of $[Fe(CN)_6]^{3-}/[Fe(CN)_6]^{4-}$ from solution and thus suppressed the Seebeck effect.

Supplementary Note 3

The effect of amide species on S_e: Amide derivatives such as urea are powerful hydrogen bond donors/accepters. They can form intramolecular hydrogen bonds or intermolecular hydrogen bonds with water molecules. Highly soluble urea enhances the Seebeck effect in the $[Fe(CN)_6]^{3-}/[Fe(CN)_6]^{4-}$ electrolyte. To clarify the underlying mechanism, we further evaluated the enhancement effects of six other amide derivative species (**Supplementary Table1**). Similar to urea, the highly soluble amide species, including acrylamide, propanamide and formamide, all enhanced the Seebeck effect. However, the slightly soluble amide species, including thiourea, biuret and hydroxycarbamide, had little effect. The highly soluble amide species are prone to form strong intermolecular hydrogen bonds with water, whereas the slightly soluble amide species tend to form intramolecular hydrogen bonds. Therefore, we propose that highly soluble amide species enable the rearrangement of hydration shells for $[Fe(CN)_6]^{3-}/[Fe(CN)_6]^{4-}$, thus resulting in the increase in S_e.

In contrast to urea, the composites of other highly soluble amide species with GdmCl did not synergistically enhance the Seebeck effect in the $[Fe(CN)_6]^{3-}/[Fe(CN)_6]^{4-}$ electrolyte (**Supplementary Figure 7**). S_e gradually decreased with increasing concentrations of these amide species, including acrylamide, propanamide and formamide, in the electrolyte containing GdmCl at 2.6 mol L^{-1}. Urea tends to form the stronger hydrogen bonds with $[Fe(CN)_6]^{3-}$, whereas GdmCl tends to bond with $[Fe(CN)_6]^{4-}$; these differences in affinity synergistically increase the entropy difference between the redox couple and thus enhance the Seebeck effect. In contrast to urea, other highly soluble amide species are more apt to bond with $[Fe(CN)_6]^{4-}$ than with $[Fe(CN)_6]^{3-}$. Thus, these amide species can compete with GdmCl to arrange the solvation shells of $[Fe(CN)_6]^{4-}$, resulting in an antagonistic effect for enhancing S_e in the $[Fe(CN)_6]^{3-}/[Fe(CN)_6]^{4-}$ electrolyte.

Supplementary Note 4

The thermal conductivity measurement by a steady-state method: The thermal conductivity of the TGC systems was measured by a steady-state method via a planar cell. As illustrated in **Supplementary Figure 11a-b**, the cell consisted of two polymethylmethacrylate (PMMA) square plates in parallel as the heat transfer walls, each with a thickness of 5 mm and a thermal conductivity (κ_1) of 0.16 W m^{-1} K^{-1}. A heat plate was placed in close contact with the right-hand PMMA wall. The other walls of the cell were sealed by polyethylene terephthalate thin sheets with a thickness of 0.5 mm and then covered by polystyrene foam to prevent heat exchange between these walls and the surroundings. The electrolyte with the thermal conductivity (κ_2) filled the cell. The principle of the steady-state method is based on heat-flux conservation. Namely, the heat flow across the two PMMA walls is equal to that across the electrolyte, defined as

$$\kappa_1 \times A \times \left(\frac{\partial T}{\partial d}\right)_1 = \kappa_2 \times A \times \left(\frac{\partial T}{\partial d}\right)_2 = \kappa_1 \times A \times \left(\frac{\partial T}{\partial d}\right)_3 \quad (2)$$

According to the Supplementary Equation 2, if the temperature gradient ($\partial T/\partial d$) across the PMMA walls and electrolyte is measured, we can obtain the thermal conductivity of the electrolyte. As shown in **Supplementary Figure 11c**, the temperature gradient was acquired by infrared imaging. Temperature values at different positions across the whole cell were obtained by using special software (SmartView 3.7) with the infrared imaging device. For each measurement, the device was allowed to equilibrate for sufficient time (>1 h) to ensure that a steady temperature gradient was received.

Supplementary Note 5

The performance of TGC at cold temperature: To illustrate stability at cold temperatures, three TGC systems were operated for a long time in the temperature range from 273 to 278 K. As shown in **Supplementary Figure 13**, The open-circuit voltage of ~7, 13 and 21 mV for three systems all maintained stably at a temperature difference of ~5 K during long-time operation (Supplementary Figure 13a). However, the steady-state current density of approximately 1.4 A m^{-2} for blank was significantly lower than that for GdmCl and UGdmCl, approximately 2.2 and 3.3 A m^{-2}, respectively (**Supplementary Figure 13b**). By contrast, at the room temperature, the current density for blank is comparable with that for GdmCl and UGdmCl (**Figure 3e** in the main text). This result indicates that the performance of blank system decays at cold temperature due to the crystallization of the redox ions at low temperature (**Figure 3d** in the main text). On the contrary, urea and guanidinium is proposed that can inhibit crystallization of the redox ions and confirmed the long-term stability and good performance of UGdmCl at low temperature.

Supplementary References

1. Hess, B.; Kutzner, C.; van der Spoel, D.; Lindahl, E., GROMACS 4: Algorithms for Highly Efficient, Load-Balanced, and Scalable Molecular Simulation. *J. Chem. Theory Comput.* **4**, 435-447 (2008).
2. Prampolini, G. *et al*. Structure and Dynamics of Ferrocyanide and Ferricyanide Anions in Water and Heavy Water: An Insight by MD Simulations and 2D IR Spectroscopy. *J. Phys. Chem. B* **118**, 899-912 (2014).
3. Åqvist, J. Ion-water interaction potentials derived from free energy perturbation simulations. *J. Phys. Chem.* **94**, 8021-8024 (1990).
4. Wu, Y.; Tepper, H. L. & Voth, G. A. Flexible simple point-charge water model with improved liquid-state properties. *J. Chem. Phys.* **124**, 024503 (2006).
5. Jorgensen, W. L.; Maxwell, D. S. & Tirado-Rives, J. Development and Testing of the OPLS All-Atom Force Field on Conformational Energetics and Properties of Organic Liquids. *J. Am. Chem. Soc.* **118**, 11225-11236 (1996).
6. Vanzi, F.; Madan, B. & Sharp, K. Effect of the protein denaturants urea and guanidinium on water structure: A structural and thermodynamic study. *J. Am. Chem. Soc.* **120**, 10748-10753 (1998).
7. Yeh, I.-C. & Berkowitz, M. L. Ewald summation for systems with slab geometry. *J. Chem. Phys.* **111**, 3155-3162 (1999).
8. Berendsen, H. J. C.; Postma, J. P. M.; van Gunsteren, W. F.; DiNola, A. & Haak, J. R. Molecular dynamics with coupling to an external bath. *J. Chem. Phys.* **81**, 3684-3690 (1984).
9. Yu, P.; Yang, F.; Zhao, J. & Wang, J. Hydration Dynamics of Cyanoferrate Anions Examined by Ultrafast Infrared Spectroscopy. *J. Phys. Chem. B* **118**, 3104-3114 (2014).
10. Babu, C. S. & Lim, C. Theory of Ionic Hydration: Insights from Molecular Dynamics Simulations and Experiment. *J. Phys. Chem. B* **103**, 7958-7968 (1999).
11. Parsons, D. F.; Boström, M.; Nostro, P. L. & Ninham, B. W. Hofmeister effects: interplay of hydration, nonelectrostatic potentials, and ion size. *Phys. Chem. Chem. Phys.* **13**, 12352-12367 (2011).
12. Collins, K. D. *et al*. Ions from the Hofmeister series and osmolytes: effects on proteins in solution and in the crystallization process. *Methods* **34**, 300-311 (2004).
