# Supplementary material for: Aqueous thermogalvanic cells with a high Seebeck coefficient for low-grade heat harvest
Source: Nat Commun. 2018 Dec 4;9:5146. doi: 10.1038/s41467-018-07625-9 (PMC6279834; doi:10.1038/s41467-018-07625-9)
Supplement: Supplementary file 2 — Description of Additional Supplementary Files [file 41467_2018_7625_MOESM2_ESM.pdf]

Description of Additional Supplementary Files

File Name: Supplementary Movie 1
Description: Powering LED arrays directly.

File Name: Supplementary Movie 2
Description: Mechanical properties of the modules.
